# Ease and practicability of the 2017 classification of periodontal diseases and conditions: a study of dental electronic health records

**DOI:** 10.1186/s12903-024-04385-5

**Published:** 2024-05-28

**Authors:** Muhammad Raza, Daniela Gurpegui Abud, Joseph Wang, Jaffer Ahmed Shariff

**Affiliations:** 1 Section Chief of Periodontics at Jamaica Hospital Medical Center, Jamaica, New York, NY 11418 USA; 2 Private Practice, New York, NY 11375 USA; 3 Division of Periodontics, Section of Oral, Diagnostic and Rehabilitation Sciences, College of Dental Medicine, Columbia University, New York, NY 10032 USA; 4 Touro University College of Dental Medicine at New York Medical College, Hawthorne, NY 10532 USA

**Keywords:** Periodontal disease, Classification, Retrospective study, Electronic health records

## Abstract

**Background:**

A new classification for *Periodontal and Peri-implant Diseases and Conditions* was introduced in the 2017 World Workshop. In the past the 1999 Armitage Classification was commonly used in practice. This study aimed to assess the ease and practicability of retroactively diagnosing a subset of patients formerly diagnosed using the 1999 AAP/CDC classification with the 2017 AAP/EFP disease classification.

**Methods:**

A random subset of 10% of all patients referred over a 7-year period (2011–2018) to the Post-Doctoral Periodontics Clinic at Columbia University College of Dental Medicine were reviewed by accessing the Electronic Health Records (EHRs) on axiUm. Patients diagnosed with periodontal disease based on the 1999 AAP/CDC classification (including *chronic* and *aggressive* Periodontitis) were reclassified using the 2017 classification (stage: I, II, III and grade: A, B, C).

**Results:**

A sample of 336 patient records were examined. 132 were diagnosed with gingivitis, and 204 with periodontitis. Of these 204 patients, 68 (33.3%) were diagnosed with aggressive and 136 (66.7%) with chronic periodontitis. Patients diagnosed with aggressive periodontitis, 10% were reclassified as stage II, 47% as stage III, and 43% as stage IV periodontitis, and 100% were reclassified as grade C. Among patients with *chronic* periodontitis, 7% were reclassified as stage I, 65% as stage II, 21% as stage III, and 7% as stage IV; 11% of these were reclassified as grade A, 63% grade B, and 26% grade C.

**Conclusions:**

The majority of those originally diagnosed with *aggressive* (90%) and *chronic* (80%) periodontitis were reclassified as either molar/incisor pattern stage III grade C or stage IV grade C periodontitis, and stage II or III periodontitis, respectively. The study demonstrated that it is practical to retroactively reassign a diagnosis according to the new 2017 classification using available information included in dental EHRs.

## Learning objectives

To understand the importance of properly documenting clinical data in the Dental setting.

To further understand the 2017 Periodontal Disease Classification and the significance of the staging and grading systems.

To determine what clinical parameters should be recorded and to discuss how to use them to assign staging and grading and accurately diagnose our patients correctly.

To recognize the practicability of retroactively applying the 2017 Periodontal Disease Classification criteria to patients formerly diagnosed using the 1999 classification to improve patient care.

To assess clinical data and determine whether surgical treatment is recommended for the treatment of periodontal disease.

## Introduction

Several periodontal classifications have been introduced over the past decades, including the 1989 World Workshop, the 1993 European Workshop, and the 1999 Armitage Classification [1], followed by the 2015 Task Force [2]. A new classification for *Periodontal and Peri-implant Diseases and Conditions* was introduced in the 2017 World Workshop and for the first time conjointly by the American Academy of Periodontology (AAP) and the European Federation of Periodontology (EFP) to address some of the diagnostic issues seen in the previous classifications. This classification proposes a multi-dimensional diagnostic approach by introducing a *staging* and *grading* system similar to that used in Oncology, where staging is based on the severity of the disease and the complexity of the case management, and grading addresses biological features such as the rate of disease progression and the risk of further advancement and potential threats to general health, to estimate chances of survival. In the new classification, and for the first time, the *staging* and *grading* system allows the clinician to use the patient’s medical and dental health records to estimate the likelihood of future bone loss with the objective of identifying high-risk patients and setting individual recall schedules to avoid worsening and optimize patient care [3]. 

Another significant change stemmed from the fact that after reviewing the current body of literature regarding the pathophysiology of periodontitis, the Workshop concluded that there was not enough evidence to support that *chronic* and *aggressive* periodontitis have individual characteristic features and are caused by different pathogens; hence, they should not be considered 2 separate diseases. Consequently, the 2017 classification eliminated the terms *aggressive* and *chronic* and divided periodontitis into (a) necrotizing periodontitis [4], (b) periodontitis as a manifestation of systemic disease [5], and (c) periodontitis [6]. 

This classification has precipitated mixed opinions among professionals, and clinicians are still learning how to implement it. Therefore, the primary purpose of this article was to retroactively diagnose a subset of patients who had formerly been diagnosed with the 1999 Centers for Disease Prevention and Control (CDC)/AAP classification [1], with the new 2017 AAP/EFP classification [6] aiming to assess the ease and practicability of assigning this new diagnosis with the information available in patients’ EHRs. Secondly, the distribution of stages and grades among patients formerly diagnosed with *chronic* or *aggressive* periodontitis will be analyzed so to further help professionals, general practitioners, and specialists implement this diagnosis in their daily practice.

## Materials and methods

### Inclusion & exclusion criteria

To be included, patients must had been referred to and seen in the Periodontics Clinic at Columbia University College of Dental Medicine (CU-CDM) between June 1st, 2011, and June 30th, 2018. A complete medical history, a Periodontics specialty consult that included a periodontal diagnosis, and a diagnostic set of radiographs were the inclusion criteria for this study. A full-mouth series taken within one year of being seen in the Periodontics Clinic was required for adults (≥25 years old), and four bitewings along with two periapical radiographs of the maxillary and mandibular anteriors were the requirements for children (< 18 years old) and young adults (18 to 25 years old). Radiographs had to be of good diagnostic quality, where the alveolar crest of bone was clearly visible, and the entire root length was captured.

Patients without this information or having low-quality or non-diagnostic radiographs were excluded.

### Study design

**Fig. 1:**
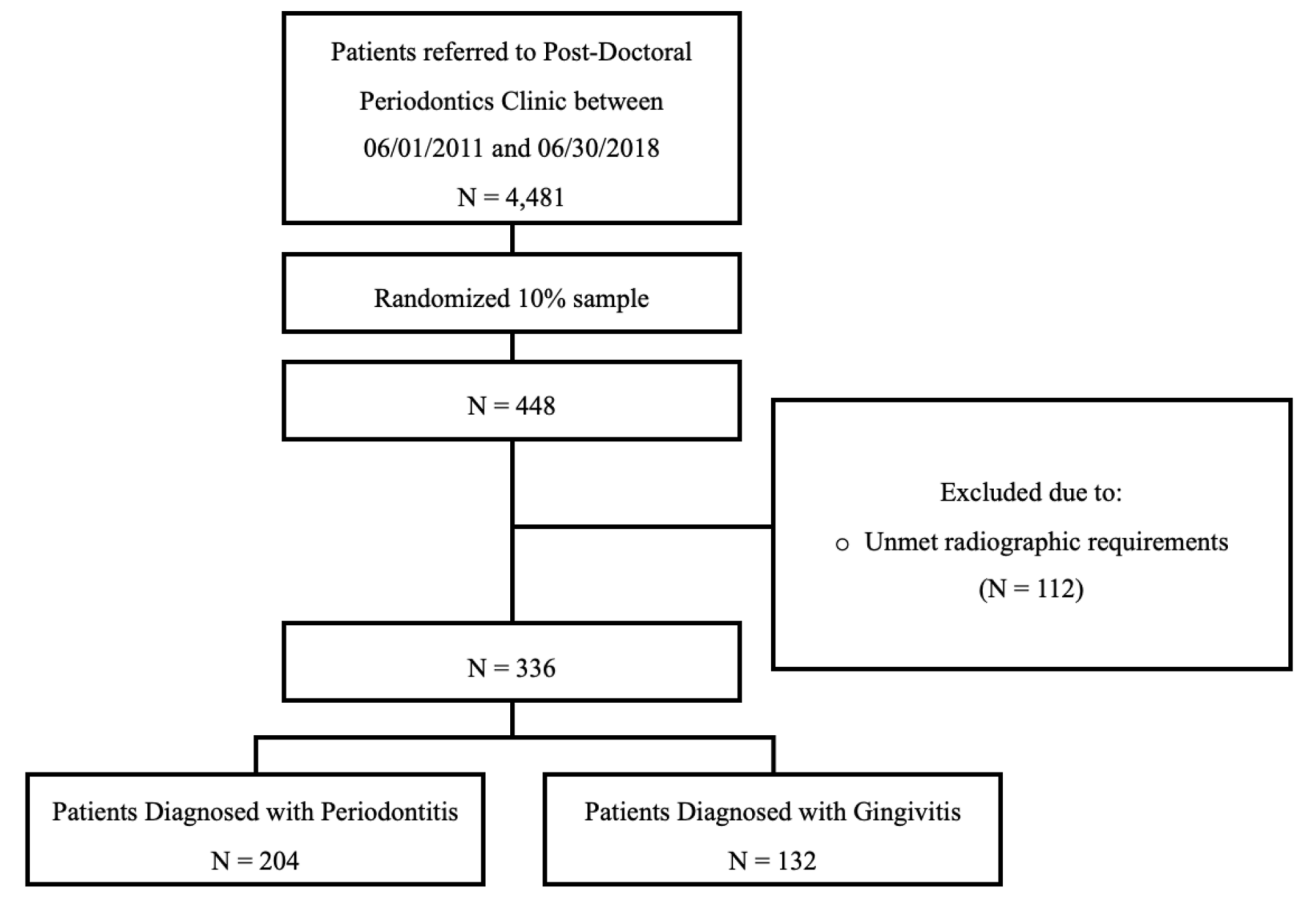
Flowchart of patient enrollment

This retrospective study reviewed the EHRs of the axiUm Patient Management System (Henry Schein) used at the Dental school during the aforementioned time period. As noted in Fig. 1, a random subset of 10% out of the 4,481patients referred to the Periodontics Clinic at CU-CDM from June 1st, 2011, to June 30th, 2018, was obtained, and a total of 448 patients were selected. 112 patients were excluded due to unmet criteria, and the remaining 336 constituted the final sample, of which 204 were diagnosed with periodontitis and 132 with gingivitis.

A single independent reviewer (M.R.) reviewed the EHRs of patients, including demographics, medical and dental histories, existing radiographs, and periodontal charts of all included patients to assign a diagnosis, according to both the 1999^1^ and the 2017^6^ periodontal disease classifications. Inter-rater reliability among three experts (J.A.S., D.G.A, J.W.) was calculated using percentage agreement for the diagnosis of 10 random patients to define the “gold standard.” Then, inter-rater reliability between the “gold standard” and the independent reviewer (M.R.) was calculated using the same methodology (10 random patients and percentage agreement).

### Data collection

Diagnosis as per the 1999^1^ classification was extracted from EHRs, and cases were divided into aggressive [7] and chronic periodontitis [2]. This classification mainly uses radiographic bone loss (RBL) and the patient’s periodontal chart for the diagnosis of periodontitis [8]. As recommended by the 2015 Task Force [2], rapid bone loss in young individuals is characterized as aggressive periodontitis; and its extent is characterized as localized when involving first molars, incisors, and ≤ 2 teeth other than first molars and incisors, while in generalized aggressive periodontitis first molars, incisors and > 2 teeth other than first molars and incisors are involved. *Chronic* periodontitis has different severity thresholds: mild (PD > 3, < 5 mm, RBL ≥2, ≤3 mm, clinical attachment loss (CAL) 1–2 mm), moderate (PD ≥5, < 7 mm, RBL > 3 to ≤ 5 mm, CAL 3–4 mm), and severe (PD ≥ 7 mm, RBL > 5 mm, CAL ≥ 5 mm), and its extent was characterized as localized when ≤ 30% of sites are affected, and as generalized when disease involves > 30% of sites. Periodontitis diagnosis was extracted directly from EHRs, specifically from the Periodontal consults. (Fig. 1).

Assignment of diagnosis according to the 2017 classification was made following Papapanou et al., recommendations [6]. In brief, stage was mainly determined using RBL and type of bone loss (vertical vs. horizontal). RBL was used to make an initial “quick” assessment and determine whether the patient belonged in either stages I/II, where RBL ≤33% and was mostly horizontal, or stages III/IV if RBL extended to the middle third of the root or beyond and there was presence of vertical defects.

Presence of furcation involvement was used to further assess stage, where class II or higher indicated stages III/IV. Recognition of furcation involvement poses a challenge to the clinician. Radiographs are only partially reliable due to frequent overlap between roots that, in turn, obscure the furcation. Moreover, anatomical features, such as intermediate bifurcation ridges (present running mesiodistally in about two-thirds of patients), makes an accurate diagnosis rather difficult. This study included patients who had been diagnosed at the Periodontics Clinic at CU-CDM, where furcation involvement is by protocol assessed using a Nabers probe by residents and confirmed by faculty members, all of whom are expert Periodontists. Yet, due to the naturally complex anatomy of multirooted teeth, accurate diagnosis of furcation involvement is rather difficult.

Another parameter of this new classification is the number of missing teeth lost due to periodontitis. As a retrospective study, this could not be directly confirmed through anamnesis and was indirectly inferred using existing radiographs, clinical findings, periodontal charts, and patient’s oral risk assessment forms.

Conversely, grading was determined using direct (when available) or indirect evidence of disease progression and using the so-called “grade modifiers.” A default grade B was assigned to all patients and then upshifted or downshifted based on the aforementioned criteria. Evidence of no radiographic bone loss, bone loss of < 2 mm, and of ≥ 2 mm over the previous 5 years determined grades A, B, and C, respectively. Grade was assigned using indirect evidence of progression if previous radiographs were not available based on the ratio (% bone loss/age) to assign grade A if < 0.25, grade B if 0.25-1.0, or grade C if > 1.0. Information regarding diabetes and smoking status was extrapolated and evaluated as “grade modifiers,” and daily cigarette consumption (none, ≤ 10 or > 10 per day) and most current glycated hemoglobin (HbA1C) percentage (< 7 or ≥ 7%) were obtained in order to accurately determine grade.

## Results

### Inter-rater reliability

Percentage agreement between the two experts (J.A.S., J.W.) was 100% for staging, grading, and extent. Percentage agreement between the single independent reviewer (M.R.) and the “gold standard” was 90%, 100%, and 100% for staging, grading, and extent, respectively.

### Patient characteristics and distribution

A total of 204 periodontitis patients were selected based on the 1999^1^ classification, with a mean age of 42.3 years (age range 13–90 years). 120 (59%) of the patients were female; 23 (11%) of the patients were ever smokers, and 20 (15%) had diabetes mellitus (Table 1).

**Table 1:** Demographic characteristics, diabetes, and smoking status of the study population

Characteristics	Aggressive Periodontitis	Chronic Periodontitis	Total
Sample distribution (N)	68	136	204
Age [mean in yrs. (IQR)]	28.7 (13–55)	48.5 (18–90)	42.3 (13–90)
	**n (%)**	**n (%)**	**n (%)**
Sex			
◾ Female	39 (57%)	81 (60%)	120 (59%)
◾ Male	29 (43%)	55 (40%)	84 (41%)
Diabetes	N/A	20 (15%)	20 (15%)
Smoking Status			
◾ Ever Smoker	8 (12%)	15 (11%)	23 (11%)
◾ Current < 10 cigarettes	2 (3%)	4 (3%)	6 (3%)
◾ Current ≥ 10 cigarettes	4 (6%)	7 (5%)	11 (5%)
◾ Unknown no. of cigarettes	2 (3%)	4 (3%)	6 (3%)

IQR: Inter Quartile Range

### Reclassification of aggressive periodontitis according to the 2017 classification

After reclassifying patients from former diagnosis of *aggressive* periodontitis using the 1999^1^ classification to the 2017^6^, none of the patients with *aggressive* periodontitis were classified as stage I, 7 (10%) patients were classified as stage II, 32 (47%) as stage III, and 29 (43%) as stage IV periodontitis. Concerning grade, 100% of these patients were classified as grade C, primarily based on the % bone loss/age ratio. Four patients qualified as grade C due to smoking (≥ 10 cigarettes/day) habit.

### Reclassification of chronic periodontitis according to the 2017 classification

Amongst the 136 patients diagnosed with *chronic* periodontitis as per the 1999^1^ classification, the majority fell into either stage II (*n* = 88; 65%) or III (*n* = 29; 21%), while the remaining 19 patients were almost equally distributed between stages I and IV. With regards to grading, 11% of these patients were classified as grade A, 63% as grade B, and 26% as grade C. Of the 35 patients that qualified as grade C, 10 (29%) patients did so due to heavy smoking and 3 (8.6%) due to uncontrolled controlled diabetes. In addition, the percent bone loss/age ratio for these patients was > 1 for all those patients, and indirect evidence of progression would have been enough to grade them accurately (Table 2).

**Table 2:** Distribution of patients formerly diagnosed as per the 1999 classification with *aggressive* and *chronic* periodontitis, according to the 2017 classification

Stage	Grade	Aggressive Periodontitis (*n* = 68)	Chronic Periodontitis (*n* = 136)	Total (*n* = 204)
I	A	-	5 (4%)	5 (2%)
	B	-	4 (3%)	4 (2%)
	C	-	-	-
II	A	-	10 (7%)	10 (5%)
	B	-	65 (48%)	65 (32%)
	C	7 (10%)	13 (10%)	20 (10%)
III	A	-	-	-
	B	-	14 (10%)	14 (7%)
	C	32 (47%)	15 (11%)	47 (23%)
IV	A	-	-	-
	B	-	3 (2%)	3 (1%)
	C	29 (43%)	7 (5%)	36 (18%)

### Distribution of periodontitis according to the 2017 classification

Overall, stage II grade B periodontitis was the most common diagnosis (*n* = 65, 32%), followed by stage III grade C periodontitis (*n* = 47, 23%) and stage IV grade C periodontitis (*n* = 36, 18%). No patients were diagnosed with stage III grade A or stage IV grade A periodontitis (Table 2.).

## Discussion

In this retrospective study, data from EHRs at a U.S. dental school was reviewed to independently assign a diagnosis according to the new 2017 classification [6], using the newly developed staging and grading systems [3], to patients who had been formerly diagnosed with the 1999^1^ periodontitis classification to examine the practicality of diagnosis conversion and the distribution of severity and extent against each of the classifications.

When analyzing patient distribution, most patients were diagnosed with stage II grade B periodontitis, as seen in Table 2. This is in contrast with Graetz et al., who found that most patients were classified as stage III grade C Periodontitis [9]. 

In accordance with previous studies, all *aggressive* periodontitis patients were assigned grade C and reclassified mostly as stage III and IV periodontitis [9, 10]. *Aggressive* periodontitis patients are individuals with severe destruction and a high probability of disease progression, also true for grade C, and as such, these results should be indication that *aggressive* periodontitis had been properly assigned, and that the grading system is a valuable tool to identify high-risk patients. To reflect this rapid and severe form of disease, the new classification refers to localized *aggressive* as “molar/incisor pattern.” Identification and early diagnosis of these patients is essential, as they require more intensive therapies, namely pocket reduction surgery, the use of local or systemic antimicrobials [10], and close monitoring with an increased frequency of maintenance to avoid future loss of teeth, aiming to preserve the patient’s function. Given the destruction and complexity of the case management in these situations (reflected in the new classification as stages III/V grade C), these types of cases should be treated ideally by Periodontists. General Practitioners and other Specialists that may encounter these patients are encouraged to promptly proceed with a referral for patients and, given the strong hereditary component, do so for siblings and children (if any) as well.

The presence of furcation involvement should always be documented during clinical examination to properly treat and manage periodontal disease. Diagnosis is challenging, and dental providers are encouraged to use adequate instruments, namely Nabers probe, to most accurately do so. Experience has been shown to positively influence the accuracy and reproducibility of the clinician’s diagnostic performance, and so providers should aim to improve their diagnostic skills in this regard and assess furcation involvement in every patient [11]. 

Similarly, radiographs of good diagnostic quality are indispensable for the diagnosis of periodontal disease. The frequency of radiographic examinations should be determined individually by assessing patient’s diagnosis (stage and grade) and overall oral risk assessment. For a patient diagnosed with either stage III or IV, with evidence of radiographic bone loss in a short time period, who is also a heavy smoker and an uncontrolled diabetic, the practitioner may choose to take radiographs every 6 months in order to confirm or rule out progression. On the other hand, if a patient is diagnosed with stage I, with no evidence of bone loss in the past 5 years and a noncontributory medical history, radiographic examination every 1 or 2 years may suffice.

The 2017 classification added a novel parameter that inputs the number of teeth lost due to periodontal disease into patient’s staging. To evaluate this, the provider may use previous dental history records. If not available, clinicians may choose to rely on patients reporting. The authors would like to indicate that recall bias may be an issue, and they encourage providers to make an educated guess with regard to the reason for tooth loss using patients’ clinical data as well. For example, if a 40-year-old male who you are seeing for the first time without previous records presents with PPD ranging from 3 to 5 mm, in which more than 30% of the sites PPD of 5 mm, 30% radiographic bone loss, with low caries risk, no history of trauma, and has lost 4 teeth (different than third molars, with no history of Orthodontic treatment), one may infer that teeth were probably lost due to periodontal disease. Thus, pushing the diagnosis from a stage II to a stage III periodontitis. This “real-life scenario” where patients are unaware of the reason behind their extraction(s) is a very common instance in the Dental Practice and one the provider encounters daily. As explained above, clinical judgment should be used to determine cause of tooth loss to properly diagnose periodontitis. It is also worth noting that “tooth loss due to periodontal disease” is only one of the many clinical parameters used in the staging system and therefore, not essential, but rather recommended, to provide a diagnosis.

To diagnose periodontitis using the 1999^1^ classification, documentation of CAL, PPD and RBL was required. Recording of these periodontal clinical parameters is highly recommended, and all practitioners should aim to do so, as they turn to be determining factors when diagnosing cases that may be in the “gray area” or between stages. However, obtaining CAL requires the documentation of PPD and location of the free gingival margin with respect to the cemento-enamel junction, which is time consuming. The reality of any busy practice, General and that of Specialties other than Periodontics, makes it problematic to do so for every patient, and so the new classification allows to use at minimum RBL and PPD, without necessarily recording CAL, along with type of bone loss (horizontal vs. vertical), mobility and furcation involvement, to predictably assign stage in most cases. The authors would like to mention that when documented correctly, these variables should suffice to provide the vast majority of patients with a diagnosis as per the new 2017 classification.

Dental clinical data that includes information with regards to CAL, as previously mentioned, is difficult to obtain. Hence, the convenience of using RBL and PPD to diagnose periodontitis is something that future researchers could use this to their advantage to help ease the study of periodontal disease. Investigators would like to, however, advise to do so with caution, as the use of all CAL, PPD, and RBL, for the diagnosis of periodontitis remains to be the most accurate method to do so. And, in these instances where information is lacking, other “complexity factors,’ like type of bone loss (horizontal vs. vertical), mobility, and furcation involvement, could be used to either upshift or downshift staging.

It is also important to note that when diagnosing periodontal disease, it is essential to differentiate between bone loss caused by periodontitis and that triggered by bone remodeling. The latter is caused by malposition, prosthetic related factors, open contacts, or iatrogenic treatment, as opposed to due to periodontitis, which is bacterial induced and host-mediated. Clinicians should subsequently carefully examine radiographs and clinical findings to rule out local factors that may have contributed to bone loss [12]. 

The extrapolated data showed that patients with stage II received either non-surgical or surgical therapy, while patients diagnosed with stages III and IV received mostly surgical treatment to restore periodontal health. As previously mentioned, diagnosis is key for the treatment and management of periodontitis, and staging and grading should be used as guidelines in their determination.

As described above, it is clear that proper documentation of clinical data is crucial to diagnose our patients. In turn, a precise diagnosis is fundamental to appropriately treat and manage periodontal disease [12]. This new classification facilitates the inclusion of several different factors to assess the severity of disease and assess the probability of future attachment loss within the patient, which greatly helps the clinician.

The conversion of the 1999^1^ classification to the new 2017^6^ classification performed for this review was overall relatively easy. Independent reviewer (M.R.) was a Periodontics resident at the time, and records were obtained from a Post-Doctoral Periodontics Clinic, as such, probably of higher diagnostic quality if compared to any Pre-Doctoral setting and maybe a General Practice [12]. Nonetheless, the authors speculate that the ease of implementation was more related to the fact that the required information (RBL, PPD, CAL, and high-quality radiographs) was readily available in patient’s EHRs, and assigning a diagnosis was a matter of “following the grid.” Thus, if data recording is well completed during clinical examination and anamnesis, the implementation of this new classification should be attainable to all practitioners.

## Conclusion

Our results demonstrate the practicability and ease of converting the 1999^1^ classification diagnoses to the new 2017^6^ classification system. *Aggressive* periodontitis translated into grade C in all instances, emphasizing that these patients’ treatment and maintenance are critical for managing their oral health. In addition, a correct diagnosis of stage III/IV Grade C periodontitis is essential as these patients require referral to the specialist for more complex care. The new system allows for a more granular description of the patient’s periodontal status and risk of progression, further aligning it with principles of personalized medicine.

Clinicians should be aware of their limitations in terms of managing a periodontally complex case, such as stages III/IV. Proper diagnosis will allow the practitioner to suggest appropriate treatment plans and adequately manage disease. An early diagnosis is extremely important to improve periodontal health and be able to avoid future tooth loss, and authors would like to encourage all practitioners, Generalists, and Specialists, to implement the new 2017 classification in order to do so.

## Data Availability

The data used to support the findings of this study are available from the corresponding author upon request.
